# Multipoint-Detection Strain Sensor with a Single Electrode Using Optical Ultrasound Generated by Carbon Nanotubes

**DOI:** 10.3390/s19183877

**Published:** 2019-09-09

**Authors:** Won Young Choi, Hyeong Geun Jo, Soo Won Kwon, Young Hun Kim, Joo Young Pyun, Kwan Kyu Park

**Affiliations:** Department of Mechanical Convergence Engineering, Hanyang University, Seoul 04763, Korea

**Keywords:** photoacoustic, multipoint detection, carbon nanotube, stretchable, strain sensor

## Abstract

With the development of wearable devices, strain sensors have attracted large interest for the detection of human motion, movement, and breathing. Various strain sensors consisting of stretchable conductive materials have been investigated based on resistance and capacitance differences according to the strain. However, this method requires multiple electrodes for multipoint detection. We propose a strain sensor capable of multipoint detection with a single electrode, based on the ultrasound pulse–echo method. It consists of several transmitters of carbon nanotubes (CNTs) and a single polyvinylidene fluoride receiver. The strain sensor was fabricated using CNTs embedded in stretchable polydimethylsiloxane. The received data are characterized by the different times of transmission from the CNTs of each point depending on the strain, i.e., the sensor can detect the positions of the CNTs. This study demonstrates the application of the multipoint strain sensor with a single electrode for measurements up to a strain of 30% (interval of 1%). We considered the optical and acoustic energy losses in the sensor design. In addition, to evaluate the utility of the sensor, finger bending with three-point CNTs and flexible phantom bending with six-point CNTs for the identification of an S-curve having mixed expansion and compression components were carried out.

## 1. Introduction

Strain sensors have attracted large interest with the development of flexible and stretchable devices. They are promising for various applications such as motion sensing of wearable devices [1,2,3,4,5], smart textiles [6,7], and soft robotics (e.g., wearable [8,9], mobile [10], and medical [11,12] robots). Strain sensors are mainly based on piezoresistive and piezocapacitive characteristics. Conventional strain sensors were fabricated using metallic materials and semiconductors. In the beginning of their development, metals were extensively investigated based on piezoresistive measurements. The studies gradually expanded to semiconductors having piezoresistive, piezocapacitive, and piezoelectric characteristics [13] and to liquid conductors [14,15,16,17]. These technologies are widely used owing to their high reliabilities and convenient operations. Although conventional strain sensors have been developed by various approaches, they have common technical limitations such as low stretchability, fixed-direction sensing, brittleness of the semiconductors, and small lifetimes of liquid conduction. In this regard, recently, many studies based on stretchable and conductive materials as alternatives have been carried out to realize human-friendly devices having high softness, stretchability, and flexibility. To develop a strain sensor having a high stretchability, conductive nanoparticles have been embedded in various types of elastomers, i.e., viscoelastic polymers. These methods can be divided into piezoresistive [18] and piezocapacitive [19,20,21] methods. The sensing performance depends on the intrinsic properties of the material. Several stretchable and conductive materials have been used, including carbon nanotubes (CNTs) [22,23,24], graphene [25,26], hydrogel [27,28,29], wrinkled platinum [30], and silver nanowires [31]. Strain sensors can be used in various applications, such as finger motion sensing [32,33], motion recognition [34], human–machine interface devices [35], and breathing and speech monitoring [36].

A strain sensor based on electric characteristics (i.e., piezoresistive, piezocapacitive, and piezoelectric) requires an electrode for each sensing area. Studies using multiple electrodes have been performed for applications requiring multiple channels [36,37,38]. In this method, complicated electronics are inevitable as the use of multiple electrodes for multichannel detection is required. In this study, we propose a multipoint detection system with a single electrode. This system is based on the ultrasonic pulse–echo method. It enables strain detection of several segments using multiple transmitters and a single receiver. The acoustic transmission signals are generated by the photoacoustic effect [39]. CNTs are used as an ultrasound emitter because they are a well-known optical absorption material generating high-pressure photoacoustic signals [40,41,42,43,44]. Polydimethylsiloxane (PDMS) is used as a stretchable polymer owing to its high thermal expansion coefficient.

In this study, several optical absorption materials based on nanoparticles were considered, such as carbon black [45], metallic nanoparticles [46], graphite [47], and CNTs. Among them, multiwalled CNTs were selected as an optical ultrasound generator. Multiwalled CNTs are attracting attention owing to their optical absorption ability capable of broad-wavelength light [44]. The high thermal conductivity of CNTs enables the rapid transfer of heat generated by CNTs to the surrounding media [48]. In particular, CNT–PDMS composites are in the spotlight recently because they generate high acoustic pressure [35].

## 2. Materials and Methods

### 2.1. Operational Principle of the Photoacoustic Strain Sensor

**Figure 1:**
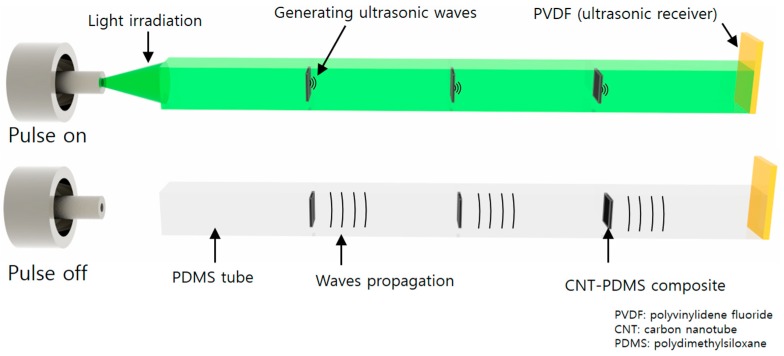
Operational principle of the strain sensor based on the photoacoustic effect.

We now present the multipoint strain detection system with a single electrode. The pulse–echo method was used with multiple transmitters and single receiver. As shown in Figure 1, transmission signals are generated by the photoacoustic effect. A photoacoustic signal is generated upon light irradiation to the optical absorption material. CNTs were used as the optical absorption material as they can emit high acoustic pressure. The stretched part consisted of PDMS, which has a high thermal expansion coefficient and stretchable characteristics. Polyvinylidene fluoride (PVDF) was used as a suitable receiver in this system because it can receive acoustic signals with a large bandwidth and a simple electrode without operation voltage. The acoustic signals generated by the CNTs at each point are delivered to the PVDF through the PDMS tube. As the speed of light is approximately 10^6^ times higher than that of sound, it can be assumed that the sound is generated at the same time. Using the received data, the CNT positions are obtained from the travel times. Therefore, the receiving times of the transmitted ultrasound signals are key factors in the strain measurement.

### 2.2. Fabrication of the Tube-Type CNT Strain Sensor

**Figure 2:**
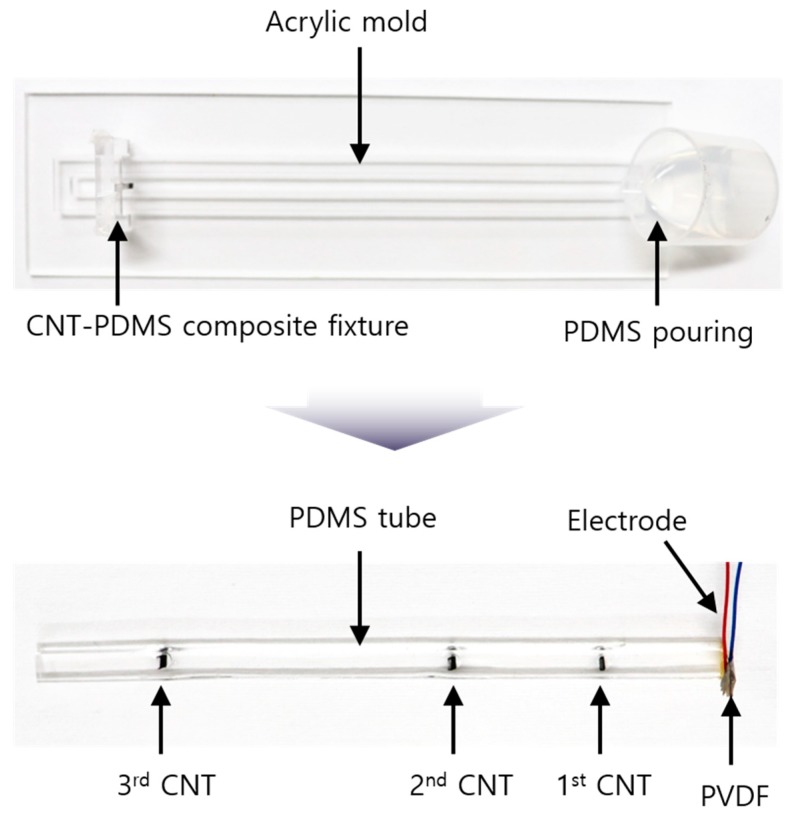
Photographs taken during the fabrication of the strain sensor.

The strain sensor was fabricated using PDMS owing to its stretchability with low optical and acoustic attenuations. The sensor was fabricated in the form of a rectangular tube to bond a large area of the surface. As shown in Figure 2, the strain sensor fabrication consisted of PDMS molding and the electrode connection of PVDF as a receiver. PDMS was mixed in a ratio of 20:1. Typically, PDMS is molded in a ratio of 10:1; however, in this study, the ratio of hardener was smaller to improve the stretching properties. The PDMS was degassed under 30 Torr in a pressure chamber with a vacuum pump (15,500, Robinair Inc., Warren, MI, USA). An acrylic structure with holes was used as a mold for the tube. The mold had a thickness of 3 mm, width of 5 mm, and length of 150 mm, i.e., the section of the tube was 3 mm × 5 mm and the length was 150 mm. Before the PDMS was poured, the CNT–PDMS composite was placed at the desired position by an acrylic fixture to arrange it perpendicular to the axial direction of tube. Since the hung composites were located parallel to the acrylic fixture, they could be placed perpendicular to the tube direction. After the PDMS pouring, the PDMS was cured on a hot plate at 65 °C for 6 h.

The electrode of the sensor was formed using a PVDF film as an acoustic receiver. PVDF was bonded to the end of the PDMS tube using a nonconductive epoxy (EPO-TEK 301, Epoxy Technology Inc., Billerica, MA, USA). The epoxy provided an excellent coupling of the PVDF and tube owing to its low viscosity. It was maintained at 65 °C for 1 h to cure. A 50 mm thin wire was bonded to the PVDF using a conductive epoxy (CW 2400, Chemtronics Inc., Kennesaw, GA, USA) to form the electrode. The wire was soldered to a simple printed circuit board for electric connection and measured using a coaxial cable.

### 2.3. Fabrication and Characterization of the CNT–PDMS Composite

**Figure 3:**
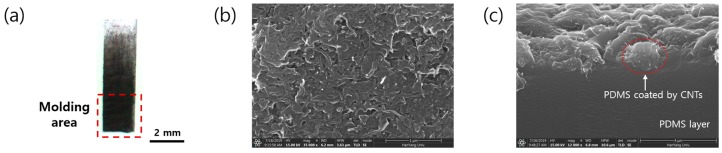
(**a**) Optical image of the CNT–PDMS composite used in the sensor; (**b**) Top-view and (**c**) cross-sectional SEM images.

It is important to generate ultrasonic pulsing signals considering the employed pulse–echo-based method. The proposed system uses the optical ultrasound generated by the photoacoustic effect as a transmission source. To generate the optical ultrasound, a material generating photoacoustic effects is required. In this study, CNT–PDMS composite was used as the CNTs (graphitized multiwall CNTs; Nanostructured & Amorphous Materials, Inc., Katy, TX, USA) have excellent optical absorption characteristics, while the PDMS has a large volume thermal expansion coefficient [49]. As shown in Figure 3a, a thin CNT–PDMS composite was fabricated by the brush-touch method using antibacterial cotton-tipped swabs. A dense coating (outlined by the dashed square) was molded into the PDMS tube. The CNT–PDMS composite had a PDMS thickness of 0.4 mm and CNT thickness of approximately 1 μm. The molded area was 2 mm × 2 mm. The cross-sectional area of the CNTs was approximately 27% that of the PDMS tube, i.e., 27% of the light energy was absorbed by the CNTs and converted to sound energy; the residual light was delivered to the CNTs behind. Figure 3b,c shows top-view and cross-sectional scanning electron microscopy (SEM) images of the CNTs coated on the PDMS, respectively.

**Figure 4:**
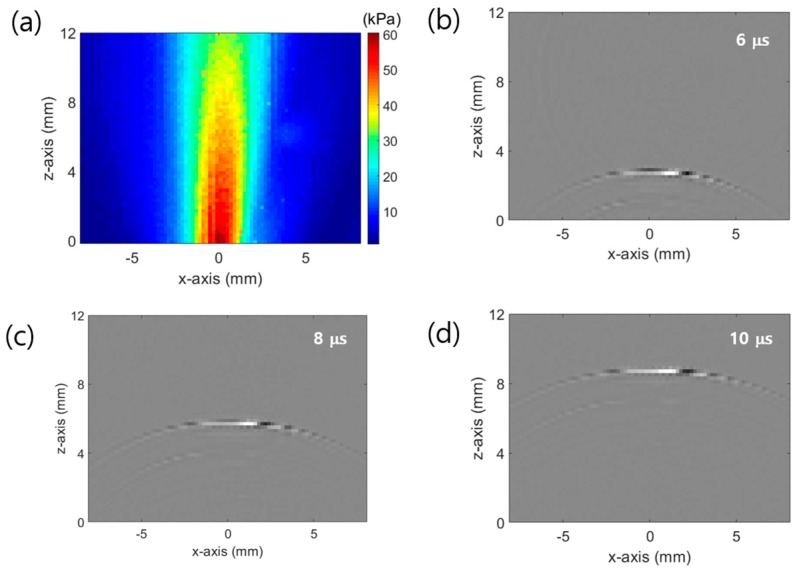
(**a**) Measured optical ultrasound pressure field generated by the CNTs in water medium; Sound wave propagation images at (**b**) 6, (**c**) 8, and (**d**) 10 μs.

The acoustic pressure was measured to evaluate the sound pressure level and wave propagation. The measurement was performed in water using a hydrophone (HNR0500, ONDA Corp., Sunnyvale, CA, USA). The employed motorized stage (SM3-0820-4S, Sciencetown Inc., Incheon, Korea) having a step resolution of 0.1 μm and uni-repeatability of 1.5 μm scanned an area of 20 mm × 20 mm with a scanning step of 0.2 mm. The measured data were calibrated by the receiving sensitivity. Figure 4a shows the pressure field of the CNT–PDMS composite used in the strain sensor, obtained using the maximum values of the enveloped measured data. The pressure field was obtained at a height of 10 mm from the CNT–PDMS composite.

The estimated light power irradiated to the CNT–PDMS composite was 0.65 mJ/cm^2^, and the maximum measured acoustic pressure was 60.5 kPa. The acoustic signal had a center frequency of 5 MHz and a 3 dB bandwidth of 96.68%. The calculated angle of directivity of the beam propagation was 4.3°. The small angle of directivity of the waveform indicates transmission in plane waves. Figure 4b–d shows the wave propagation at times of 6, 8, and 10 μs, respectively. The calculated wave velocity was 1475 m/s, similar to the reported speed of sound in water.

### 2.4. Experimental Setup

**Figure 5:**
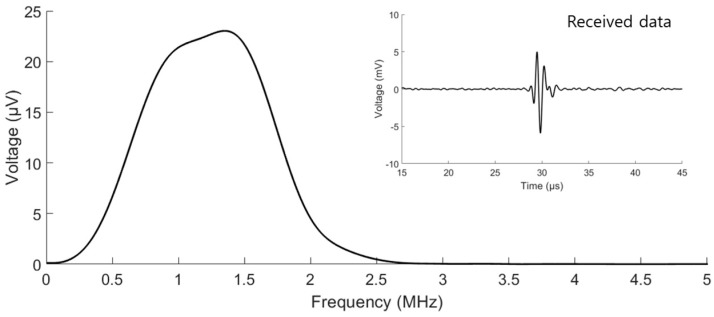
Fast-Fourier-transformed and received data generated by the strain sensor.

The fabricated strain sensor was operated using support equipment. The system setting for the operation of the strain sensor was divided into two parts: acoustic transmission and receiver parts. Light energy is needed to generate ultrasound signals by the CNT–PDMS composite. For the transmitting part, a neodymium-doped yttrium aluminum garnet pulsed laser (Minilite I, Amplitude Inc., Milpitas, CA, USA), having a wavelength of 532 nm, pulse duration of 7 ns, and pulse repetition frequency of 10 Hz, was used. The light generated by the laser was delivered to the strain sensor by an optical fiber bundle (BF46HS01, Thorlabs Inc., Newton, NJ, USA) having a core diameter of 600 μm and wire length of 1 m. The core of the bundle was aligned with the irradiated laser by an subminiature version A (SMA) fiber adapter (SM1SMA, Thorlabs Inc., Newton, NJ, USA). The transmitted ultrasonic signals were detected by the PVDF. The received signal was amplified by a preamplifier (5678, Olympus Inc., Tokyo, Japan) having a bandwidth of 200 kHz to 40 MHz and fixed-voltage gain of 40 dB and then measured by an oscilloscope (DSOX 2004A, Keysight Technologies Inc., Santa Rosa, CA, USA), with 5000 points and a sampling frequency of 500 MHz, as an average of eight acquisitions. The digital data of the oscilloscope were stored by MATLAB. As shown in Figure 5, the received data had a center frequency of 1.35 MHz and 3 dB bandwidth of 68.3%.

### 2.5. Empirical Analysis of the Energy Loss

**Figure 6:**
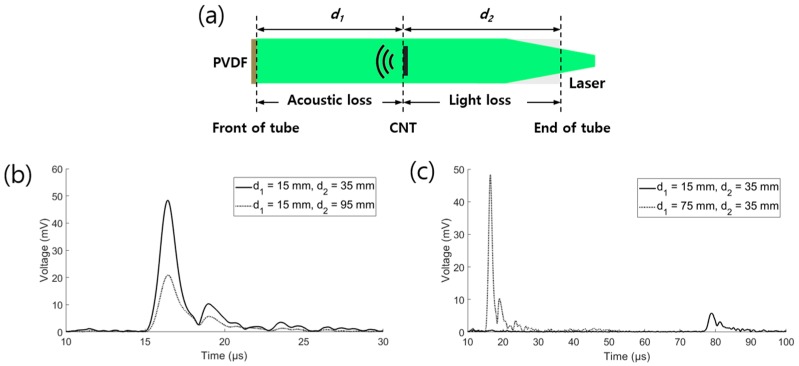
(**a**) Sensor structure used in the experiment; Enveloped received data for the same (**b**) *d*_1_ and (**c**) *d*_2_.

In the design of the strain sensor, the energy loss in the tube should be considered. Two types of energy transfer exist inside the tube: optical and acoustic. The optical waves should be transferred to the CNTs, while the acoustic waves should be transmitted from the CNTs to the PVDF. In this wave propagation, energy loss occurs in the PDMS tube. An experiment was carried out to estimate the energy loss. As shown in Figure 6a, the distance in the tube was divided into the lengths from the PVDF to the CNTs and from the CNTs to the end of the tube. Figure 6b shows the effect of the optical loss evaluated by equalizing the distance between the PVDF and CNTs, while Figure 6c shows the effect of the acoustic loss by equalizing the distance between the CNTs and the end of the tube. The pressure reduction factors over the distance of 60 mm (Figure 6b,c) were 2.32 and 8.36, respectively. The calculated optical loss was 0.192 dB/mm, while the acoustic loss was 0.301 dB/mm. Considering the optical absorption by the CNTs (Section 2.3), the ideal interval between the CNT points to equalize the amplitudes of the received signals in the strain sensor was 25.1 mm.

## 3. Results and Discussion

### 3.1. Multipoint Expansion Experiment

**Figure 7:**
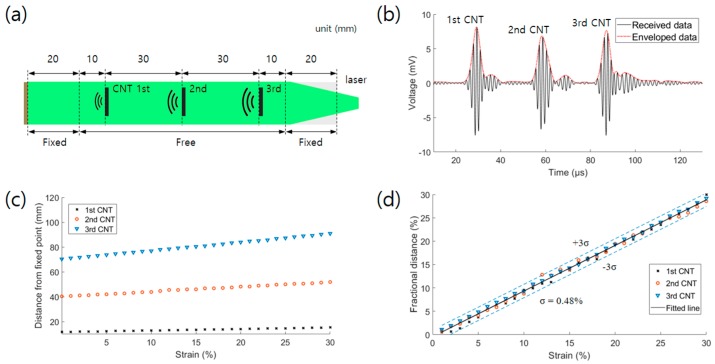
(**a**) Sensor structure used in the multipoint expansion experiment; (**b**) Received and enveloped data before the stretching; (**c**) Data of the transmission time from the initial point of the free range of the sensor according to the strain up to 30% (intervals of 1%); (**d**) Converted data from (**c**) to fractional time changes.

The main aim of this study was to implement a strain sensor system capable of multipoint strain detection with a single electrode. A strain sensor expansion test was carried out to verify the feasibility of multipoint detection. The fabricated strain sensor for the expansion test shown in Figure 7a had a spacing of 30 mm between the CNT points. The sensor was fixed by clamps having widths of 20 mm and stretched by the motorized stage. The clamped area was fixed, while the 80 mm area between the clamps was tensioned. The sensor was stretched up to a strain of 30% in intervals of 1%. Figure 7b shows the three ultrasound signals generated by the CNTs. The envelope detection processing was used to evaluate the absolute pressures of the waveforms. To analyze the shift time, the times at the maximum peaks (*T*_0_ (transmission time from the initial point of the free range of the sensor)) were extracted (Figure 7c). Figure 7c presents the increase in time from the fixed point according to the location of the CNTs. The time change increased with the distance of the CNTs from the PVDF, as expected. The fractional time change ((*T* – *T*_0_)/*T*_0_) was calculated to analyze the strain variation of each point, where *T*_0_ is *T* before the stretching. Figure 7d shows the fractional time change at each point and fitted line. The standard deviation was 0.48%. These results indicate that the strain sensor can detect axial tension.

### 3.2. Glove Bending Experiment

**Figure 8:**
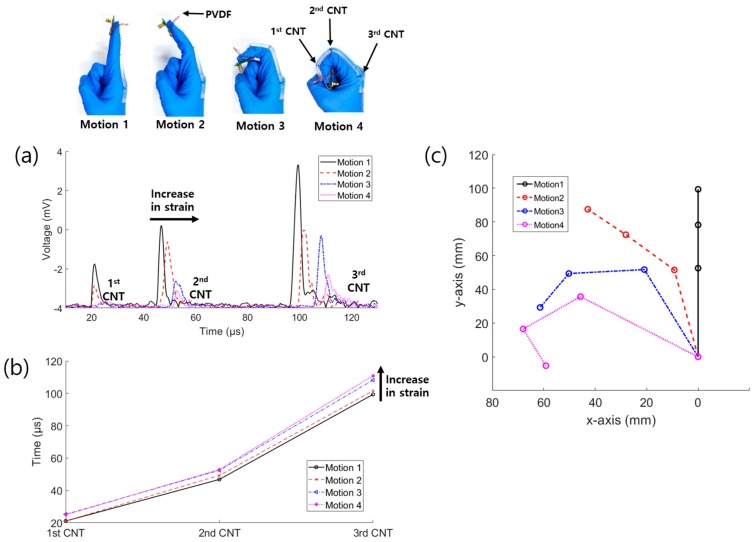
(**a**) Enveloped data according to the finger bending motion; (**b**) Corresponding times of the peak positions in (a); (**c**) visualization graph of the finger motion structure.

Further, we demonstrated the applications of the sensor for human motion detection by carrying out a finger bending experiment. The finger is divided into three segments: starting near the body, there are the proximal, middle, and distal phalanges. The device was fabricated by attaching the three-point strain sensor to a nitrile glove. The PVDF was located at the fingertip, while the three CNTs were aligned with the side of the phalangeal joint for the detection of each segment, as shown in Figure 8. The first, second, and third CNTs detected the lengths of the distal, middle, and proximal phalanges, respectively. As shown in Figure 8a, when a larger bending force was applied, the detection time of the signal increased due to the axial strain. At the corner of the bent tube, as the reflection angle was larger, a higher light energy loss by transmission occurred and the acoustic waves overlapped. Consequently, the intensity of the signal was dynamically reduced, while the pulse length was increased due to the bending. The data can be analyzed by the changes in time, intensity, and wavelength. In Figure 8b, we represented the data by the time shift, based on the basic principle of the sensor. The shifted times of Motions 2–4 compared to those of Motion 1 are presented in Table 1. The time differences were used to predict the positions of the phalanges. The results in Figure 8b obtained by peak detection show that the strain sensor can distinguish the bending motion. In finger bending, the length of the phalanges becomes longer as the finger angle increases, so the measured time can convert the joint angle. As shown in Figure 8c, the finger structure was reconstructed by estimating the joint angles. The angles were calculated by an exponential function which was acquired by fitting the angles of a real finger sample (0, 45, 90 degrees). As a result, the graph shows that the display of finger motion is possible.

### 3.3. Flexible Phantom Bending Experiment

**Figure 9:**
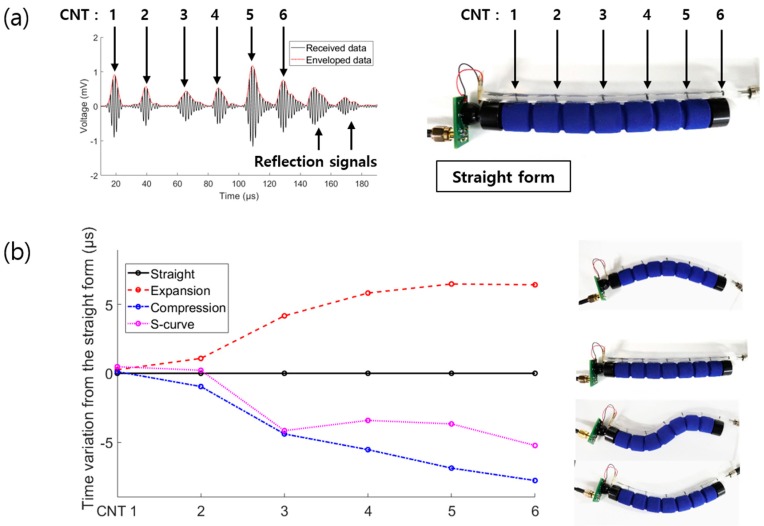
(**a**) Extracted data from the strain sensor in the straight form; (**b**) Time variations of the other forms from the straight form.

We demonstrated the identification of an S-curved form, which includes not only expansion but also compression of the sensor. To validate the S-curve bending detection using this technique, the strain sensor was attached to a flexible phantom. The strain sensor consisted of six-point CNTs placed with spacing of 20 mm in the compression form. Figure 9a shows the CNT positions of the sensor attached to the flexible phantom and the signals detected by the six-point strain sensor. The generated optical ultrasound waves in the tube were transmitted in both directions, giving frontward and backward propagations. The frontward waves were directly delivered to the PVDF, whereas the backward waves were reflected by the PDMS–air interface at the end of the tube. The received data included the ultrasonic signals generated by each CNT and the reflection signals of backward-propagation waves. Thus, the signals generated by CNTs and reflected signals could be physically distinguished.

The experiment was performed to observe the time variations in four cases: straight form, expansion, compression, and S-curved form. Figure 9b presents the time variations from the straight form, i.e., the distance variations of the CNTs from the straight form to obtain a rough outline of the strain sensor. The time variations between the CNT points exhibited an increasing trend for expansion and a decreasing trend for compression. In the case of the S-curve form, a mixture of increasing and decreasing trends was obtained, as expected. Figure 9b indicates that the strain sensor could recognize complex shapes with mixed compression and expansion components.

## 4. Discussion

In Section 2.5, the ideal interval length of the CNT–PDMS composite was empirically estimated by analyzing acoustic and light energy loss. This shows insight into the process of determining ideal parameters of the strain sensor. Although the optimized composite interval was calculated, the strain sensors used in the experiment had other interval parameters since the main purpose in this study was to present the feasibility of applicable conditions. For finger sensing, the interval of the composite was dependent on the length of the phalangeal joint (Figure 8). Thus, the composites in the strain sensors were arranged according to the application (finger bending or flexible phantom bending).

According to changes in the structure attached to the strain sensor, it is possible to have the same length based on the axis of the tube but different bending degrees. In this case, the detected time of signals is not an important factor. When applying a strain sensor to this structure, it is necessary to analyze the amplitude and pulse length of signals. During the delivery of ultrasound and light, acoustic and optical energy losses at the tube and air interfaces should be considered. In ultrasound transmission, the acoustic reflection at the interface of PDMS and air should be calculated. The acoustic signal reflectivity is affected by the incident angle and acoustic impedance [50]. In this case, the signal reduction by bending is negligible because the acoustic impedance differences of PDMS and air are significant; thus, it is assumed that the signal is totally reflected. In light transmission, a certain amount of optical energy is lost at the PDMS–air interface. The optical reflectance and transmission are calculated by the Fresnel equation. It is calculated by simplifying to refractive index differences. The energy losses are not negligible because the difference between the refractive indices of PDMS and air is typically 1.3–1.7 times. One of the criteria for bending motion detection is pulse length changes. At the corner which is created by bending, phase differences in ultrasound waves are generated according to the transmitted angle. These phase differences cause the pulse length to be longer. Therefore, the amplitude and pulse length of signals are critical factors to distinguish the bending motion of the same length.

## 5. Conclusion

We fabricated a strain sensor capable of multipoint detection with a single electrode. The sensor consisted of PDMS as the stretchable material. It was operated using the ultrasound pulse–echo method. The ultrasound signals were generated by the photoacoustic effect using CNTs as a representative high-optical-absorption material. Ultrasonic transmitters were fabricated at several points, while a single receiver was used as the only electrode to record the data. The received data could be used to detect the positions of the transmitters by analyzing time differences.

The proposed system has various advantages over piezoresistive and piezocapacitive structures, including simple multipoint detection with a single electrode, acquisition of more analytical information (e.g., transmission time, amplitude, and pulse length of the acoustic signal), and a tube-type sensor. The changes in detection time with strain up to 30% (intervals of 1%) and the loss factor of the strain sensor were evaluated. In addition, the optical and acoustic losses per length inside the tube were evaluated based on the empirical results, and the optimized CNT spacing was obtained. To demonstrate the potential of the proposed technique, an experiment was carried out to detect finger bending motion with three-point detection and a flexible phantom test was carried out to identify the complex shape of an S-curve with compression and expansion components.

## Figures and Tables

**Table 1:** Time variations from Motion 1.

Time Variation	Motion 2	Motion 3	Motion 4
1**^st^** CNT	0.08 μs	4.14 μs	3.86 μs
2**^nd^** CNT	2.48 μs	5.64 μs	6.12 μs
3**^rd^** CNT	2.26 μs	8.86 μs	11.56 μs
